# Humoral Acute Rejection in a Kidney Transplant Recipient with Idiopathic Thrombocytopenic Purpura

**DOI:** 10.1155/2021/9933354

**Published:** 2021-04-22

**Authors:** Ana Paola Rico-Portillo, José Ignacio Cerrillos-Gutierrez, Jorge Andrade-Sierra, Alfredo Gutiérrez-Govea, Enrique Rojas-Campos, Claudia Alejandra Mendoza-Cerpa, Benjamín Gómez-Navarro

**Affiliations:** ^1^Departamento de Nefrología y Trasplantes, UMAE, Hospital de Especialidades, CMNO, IMSS, Guadalajara, Jalisco, Mexico; ^2^Unidad de Investigación Médica en Enfermedades Renales UMAE, Hospital de Especialidades, CMNO, IMSS, Guadalajara, Jalisco, Mexico; ^3^Departamento de Anatomía Patológica UMAE, Hospital de Especialidades, CMNO, IMSS, Guadalajara, Jalisco, Mexico

## Abstract

A 47-year-old male was diagnosed with chronic kidney disease (CKD) in 2011; idiopathic thrombocytopenic purpura (ITP) was also diagnosed in 2011 refractory to medical treatment and finally treated with splenectomy (2017) without relapses since that date, 5 blood transfusions, and 4 platelet apheresis in 2017. Renal transplant from a living related donor (brother), ABO compatible, crossmatch were negative, sharing 1 haplotype. Donor-specific anti-HLA antibody was negative. Graft function was stable until the 5^th^ day and graft biopsy on the 6^th^ day; thrombotic microangiopathy (TMA), C4D negative and inflammatory infiltration of polymorphonuclear leukocytes inside peritubular capillary, and anti-MICA antibodies were positive. The treatment used were plasmapheresis, intravenous immunoglobulin, and rituximab. Serum creatinine began to decrease since the 14^th^ day, and by day 33, post-RT graft function was restored.

## 1. Introduction

Idiopathic thrombocytopenic purpura (ITP) is an immune disease characterized by bleeding and thrombocytopenia [1]; the association among ITP and CKD is not common, and treatment is difficult in patients due to the risk of bleeding in hemodialysis (HD) sessions [2]. Renal transplantation in patient with ITP and CKD is especially uncommon with only 4 cases reported in the literature [2–4]. Kidney transplantation in a patient with ITP is especially challenging as poor platelet function secondary to uremia adds the risk of bleeding in addition to sensitization due to the need of transfusion in ITP crisis. The main cause of graft loss in kidney transplantation, is acute rejection (AR) [5]. We do not have reports in graft recipients with ITP regarding the presentation and evolution of AR after kidney transplant [5]. This paper shows evolution of humoral acute rejection in a kidney recipient with ITP.

## 2. Case Report

A 47-year-old male was diagnosed with CKD (stage 3B) in 2011 (unknown etiology), starts HD in November 2017, and had 3 vascular access. Hypertension since 2011 was treated with losartan and metoprolol, ITP was diagnosed in 2011, firstly treated with intravenous immunoglobulin (IG) 1 g/kg and metilprednisolone 30 mg/kg IV, and after, maintenance with prednisone 4 mg/kg dose reduction. The patient had 3 ITP relapses; first one was treated with rituximab 375 mg/m^2^, 3 doses; the second one was treated with cyclosporine 2.5 mg/kg; and the third was classified as nonresponse medical treatment, reason to be treated with splenectomy, which was done in January 2017, no associated relapses since that date. During ITP evolution 5 blood transfusions and 4 platelet apheresis were required; the last transfusion was administrated in 2017.

Kidney transplant from a living related donor (brother), ABO compatible, and crossmatch for T and B lymphocytes were negative (flux cytometry), sharing 1 haplotype. Donor-specific anti-HLA antibody was negative. During surgery, one platelet apheresis was required; approximated bleeding after surgery was 100 mL. It was decided during induction to use basiliximab (20 mg) on days 0 and 4, tacrolimus (0.12 mg/kg), mycophenolic acid (2 gr), and prednisone (1 mg/kg).

**Figure 1:**
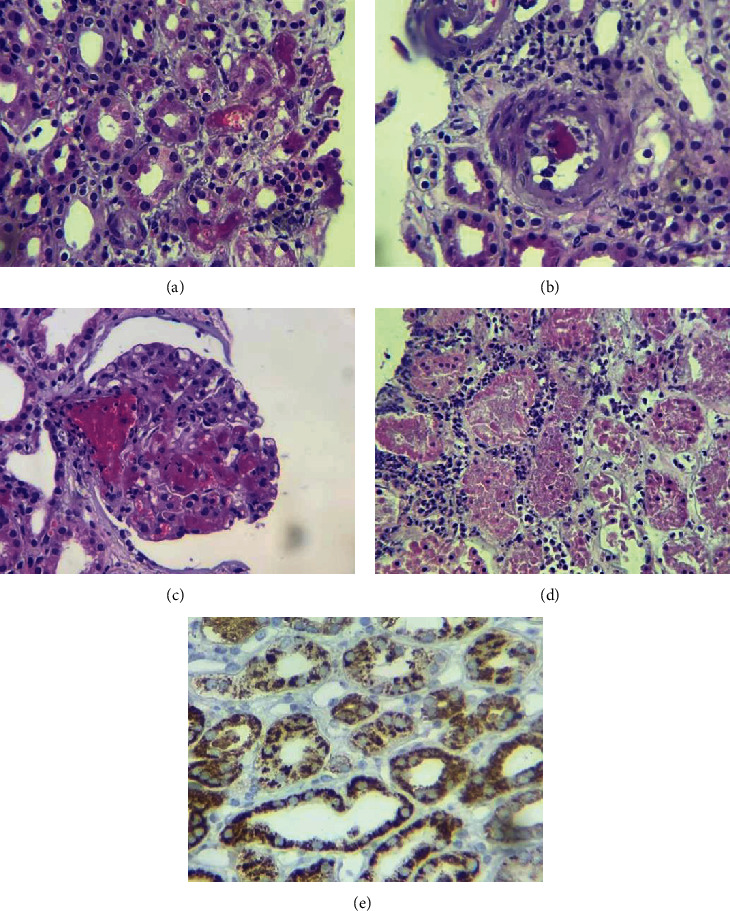
Graft biopsy: (a) microthrombosis in peritubular capillary. (b) Interstitial arteriole with a thrombus and endothelial edema. (c) Thrombosis in glomerular capillaries with retraction of glomerulus and pseudoincrease of urinary space. (d) Peritubular capillaritis due polymorphonuclear leukocytes. (H&E and magnification ×40). (e) C4d negative in peritubular capillaries (40x).

**Figure 2:**
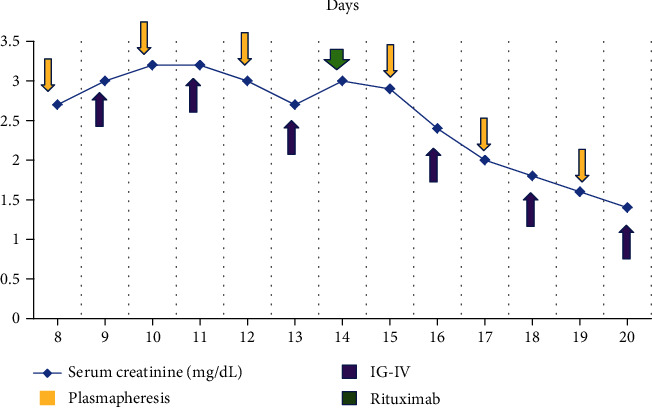
Evolution of graft function (SrCr) during antirejection treatment. Yellow arrows show plasmapheresis (days 8, 10, 12, 15, 17, and 19); green, rituximab (day 14); and purple, IG-IV treatment (days 9, 11, 13, 16, 18, and 20).

Immediate graft function was normal (Table 1), until the 4^th^ day postsurgery, serum creatinine (SCr) reaches 2.5 mg/dL, and by the 5^th^ day, SCr rises, and in the 10^th^, reached 3.2 mg/dL. In the 6^th^ day, a graft biopsy was done (previous platelet apheresis administration). Histological findings were thrombotic microangiopathy (TMA), C4D negative, and inflammatory infiltration of polymorphonuclear leukocytes inside peritubular capillaritis; humoral acute rejection was suspected based on these findings (Figure 1). Graft hematoma was found as a major biopsy complication and required 3 blood transfusions, and urology decided not to approach to surgery. A new donor-specific anti-HLA antibody test was done; negative results came on again; an anti-MICA antibody determination was positive: ∗002 9.07 MFI, MICA∗007 9.07 MFI, MICA∗009 9.07 MFI, MICA∗017 9.07 MFI, MICA∗019 9.07 MFI. Based on results and the histological pattern, a humoral acute rejection was diagnosed [6]. The treatment choices are plasmapheresis (6 doses), intravenous immunoglobulin (6 doses 0.2 mg/kg), and in the middle (between 3 and 4 dose), rituximab 375 mg/m^2^ (Figure 2). Serum creatinine began to decrease since the 14^th^ day, and by the 33^th^ day, post-kidney transplant graft function was restored (Table 1); no ITP relapse was present.

## 3. Discussion

Adult ITP is a chronic disease and uncommon in males [1]. The presence of controlled ITP should not be considered as a contraindication for kidney transplant; HD has more complications and bleeding events compared to kidney transplant [4]. Splenectomy was the first-line treatment 50 years ago; now, it is needed in those with immunosuppression and medical treatment nonresponder, as in this case [7]. Patient was classified as low risk because crossmatch and donor-specific anti-HLA antibody were negative. No information was found in the association of humoral rejection in transplanted patients with ITP. In our setting, anti-MICA antibody is an uncommon determination, but the presence of TMA and inflammatory infiltration of polymorphonuclear leukocytes inside peritubular capillary leads us to determine it [5, 8]. There are another non-HLA type of antibodies as antiendothelium; in case anti-MICA were negative, they should be determined. The presence (pre- and post-kidney transplant) of donor-specific anti-HLA antibodies as anti-MICA is associated to lower graft survival. Anti-MICA has been studied in this century and was observed in recipients who had identical HLA which develop humoral rejection; MICA antigen belongs to class I major histocompatibility complex and has higher frequency of polymorphisms [9]; it has been related to other pathologies as Crohn disease and diabetes mellitus type 1. These antigens do not follow classical HLA pathway; they present antigens to T lymphocytes, and we find them in endothelial cell surface, gastro intestinal tract, fibroblasts, monocytes, keratinocytes, and dendritic cells. It is possible that the role of anti-MICA is due to their expression in endothelial cells and it is one of the primary targets in rejection [10, 11]. Antigens mentioned are capable to induce either cellular or humoral response during graft rejection [11] not knowing the specific mechanisms and unclear role of blood transfusions in their development as a probability in this case presented [12, 13]. Plasmapheresis response, IG-IV, and rituximab show positive outcomes. Speaking of this case, by the 120^th^ day, patient was stable and without evidence of acute graft failure, either ITP relapse.

## Figures and Tables

**Table 1:** Biochemical parameters and graft function evolution.

	Evolution (days)									
	Pre-TR	1	3	4	5	6	8	10	14	33
SrCr (mg/dL)	7.4	5.7	2.8	2.5	2.7	3.0	2.7	3.2	2.4	1.3
eGFR (mL/min/1.73m^2^)	7.9	10.9	25.7	29.5	26.9	23.6	26.9	21.9	31.0	62.9
Diuresis (L)	.28	.51	7.36	4.36	5.02	9.54	2.37	1.94	3.61	2.95
Hb (mg/dL)	9.4	8.7	8.6	8.8	8.5	8.5	8.8	8.2	8.0	8.3
Plt (10^3^/*μ*L)	70	96	91	64	79	76	52	43	137	114

SrCR: serum creatinine; eGFR: estimated glomerular filtration rate; L: litters; Hb: hemoglobin; Plt: platelets.
